# Emergence, global dispersal, and local adaptations of *Yersinia pestis*, the agent of plague

**DOI:** 10.1128/aem.01658-25

**Published:** 2026-03-13

**Authors:** Victoria Carcauzon, Anne Laudisoit, Philip Slavin, Steven M. Goodman, Florent Sebbane, Pablo Tortosa

**Affiliations:** 1 Université de La Réunion, Unité Mixte de Recherche Processus Infectieux en Milieu Insulaire Tropical (UMR PIMIT), CNRS 9192, INSERM 1187, IRD 249, Plateforme Technologique CYROI, Sainte Clotilde, La Réunion, France; 2 EVECO group, University of Antwerp 26660, Wilrijk, Belgium; 3 University of Stirling 7622, Stirling, United Kingdom; 4 Field Museum of Natural History 2415, Chicago, Illinois, USA; 5 Association Vahatra, Antananarivo, Madagascar; 6 University of Lille, CNRS, Inserm, CHU Lille, Institut Pasteur Lille, U1019 - UMR 9017 - CIIL - Center for Infection and Immunity of Lille 165209, Lille, France; The Pennsylvania State University, University Park, Pennsylvania, USA

**Keywords:** plague, *Yersinia pestis*, sylvatic cycle, reservoirs, vectors, maintenance

## Abstract

*Yersinia pestis*, the causative agent of plague, is a zoonotic bacterium that split at least 6,000 years ago from *Yersinia pseudotuberculosis* through plasmid acquisition, gene loss, and point mutations. These genetic events enabled a shift from the fecal-oral route to vector-borne transmission and facilitated systemic spread. In the course of its evolutionary history, *Y. pestis* has caused an unaccountable number of human outbreaks, at both regional and pandemic scales, of which three global pandemics have been documented from historical sources. The bacterium currently persists in sylvatic (enzootic) natural foci across Africa, Asia, and the Americas. Its persistence depends on the co-occurrence of reservoir hosts (mainly mammals) and competent vectors, primarily mammal ectoparasites, and their occurrence is shaped by environmental conditions. However, ecological variation among foci and limited epidemiological data still hinder our understanding of plague dynamics. This review examines the genetic evolution of *Y. pestis*, traces the bacterium’s history, including its global spread, and presents the main actors of sylvatic plague involved in the bacterium’s maintenance, with a special emphasis on the knowledge of vectors and hosts in countries that have reported cases of plague over the past decade. The global dispersal of *Y. pestis* has led to its adaptation to several distinct environments, highlighting the need for a comprehensive surveillance strategy for a better understanding of the ecological drivers of *Y. pestis* persistence and transmission within each of the geographical regions it is maintained in the natural environment.

## INTRODUCTION

*Yersinia pestis*, the causative agent of plague, was responsible for one of the deadliest zoonotic epidemics in recorded history, the Black Death (c. 1338–1353), which devastated Eurasia, the Middle East, and North Africa and decimated an estimated 40%–60% of the European and Middle Eastern population (1, 2). Genomic analyses, including ancient DNA from archeological remains, have placed the emergence of *Y. pestis* at approximately 6,000 years ago or more (3). These data, together with reverse genetics experiments, indicate that *Y. pestis* evolved from a *Yersinia pseudotuberculosis* progenitor through a series of gene losses (pseudogenization) and acquisitions via horizontal gene transfers, enabling its transition from an enteric to an arthropod-borne pathogen with enhanced systemic tropism (4).

This evolutionary shift was followed by an unknown number of epidemics, followed by three major pandemics. The scale of each pandemic’s geographical spread was closely linked to trade, the speed, and evolution of means of transportation and conflicts, ranging from Eurasia and North Africa during the first (c. 541–770) and second (c. 1338–1844) pandemics to global dissemination during the third (c. 1894–1950). In the case of the latter, *Y. pestis* was introduced to many “virgin” regions and became endemic in several, including South America, Western United States (USA), Madagascar, and South Africa (5). Today, enzootic transmission of *Y. pestis* persists in several countries, with emergence episodes (epizootics and epidemics) occurring either seasonally, as in Madagascar, the Democratic Republic of Congo (DR Congo), or after prolonged dormant periods, as in Algeria. While the evolutionary origins of *Y. pestis* and the historical pathways of pandemics are relatively well documented, its ecological persistence and the mechanisms underlying endemization remain poorly understood.

## *YERSINIA PESTIS* AND THE EMERGENCE OF PLAGUE

*Yersinia pestis* is a gram-negative, facultative anaerobic coccobacillus (1–3 µm in length), non-motile, and non-spore-forming (6). It belongs to the *Yersiniaceae* family, recently placed in the order Enterobacterales, comprising six genera (7). The genome of *Y. pestis* consists of a ~4.65 Mb circular chromosome and three plasmids. One of the plasmids, pYV (or pCD1, 70-75 kb), is shared with *Y. pseudotuberculosis*. The other two, pMT1 (or pFra, ~110 kb) and pPCP1 (or pPla, 9.6 kb), are absent in *Y. pseudotuberculosis* and were acquired through horizontal gene transfer, allowing flea-borne transmission of *Y. pestis* and lymphatic dissemination in the vertebrate host (6, 8). In addition, a number of subtle mutations resulting from local adaptations led to 33 currently acknowledged phylogroups of *Y. pestis*, distributed across five biovars (Microtus, Intermedium, Antiqua, Medievalis, and Orientalis) (9). For instance, the Microtus biovar, including 0.PE2 to 0.PE5 lineages, expresses limited virulence and has adopted a mostly enzootic lifestyle, in contrast with the other four biovars. Each phylogroup displays distinct micro-evolutionary trajectories, as illustrated by the evolution of the Pla protein, which led to moderate virulence in vertebrate hosts, thus increasing bacterial transmission (10, 11).

### Arthropod adaptation

*Yersinia pestis* adapted to new aspects associated with vector-borne transmission, such as inactivation of factors harmful to the vector, tolerance to toxic compounds in the flea digestive tract, and an ability to switch hosts while maintaining effective dispersal.

Urease is the factor harmful to the vector that has been inactivated during the emergence of *Y. pestis* (12, 13). The enzyme enables enteric bacteria to survive in acidic environments by hydrolyzing urea into ammonia and carbon dioxide, thus neutralizing the acidity typical of enteric environments. Comparison of the *ureABCDE* chromosomal loci of *Y. pseudotuberculosis* and *Y. pestis* revealed a loss of urease function in *Y. pestis*, caused by a frame-shift mutation in the gene encoding the chaperone UreD (12). This loss of function highlights the adaptation of *Y. pestis* to its vector by reducing its toxicity toward fleas, enabling the bacterium to persist without compromising vector survival, thereby increasing its chances of being maintained in the population (13).

The colonization of arthropods was further facilitated by the *Yersinia* murine toxin (*ymt*) gene, expressed by the horizontally acquired pMT1 plasmid. This gene encodes a phospholipase D whose activity protects the bacteria from digestion in the flea gut when the flea ingests mouse blood but not brown rat blood (14, 15). In other words, the horizontal acquisition of *ymt* expands the range of hosts that could support flea-borne plague (15).

Once ingested by the flea, in the proventriculus, many bacteria are retained within a soft mass of unknown origin, composed of blood meal-derived proteins and lipids (16–19). During each subsequent meal, blood flow may displace all or part of the mass, which can partially reform. This cycle repeats until the bacteria fully colonize and harden the mass (via the export of the poly-β−1,6-N-acetyl-D-glucosamine exopolysaccharide into the extracellular matrix) so firmly that it can no longer be dislodged by the blood flow (16, 20, 21). This obstruction induces a state of starvation, increasing vector aggressiveness and intensifying biting behavior toward mammalian hosts, thereby promoting the transmission of the bacillus by regurgitation.

In *Y. pestis* and *Y. pseudotuberculosis*, poly-β−1,6-N-acetyl-D-glucosamine biosynthesis and export are controlled by the *hmsHFRS* chromosomal regulon through the tuning of cytoplasmic cyclic di-GMP (22, 23). The HmsR and HmsS proteins form an inner-membrane-associated complex with glycosyltransferase activity, responsible for synthesizing the exopolysaccharide poly-β−1,6-N-acetyl-D-glucosamine. This polymer is then exported to the extracellular space by the HmsH and HmsF out-membrane proteins (23). *Yersinia pestis* forms consistently thicker biofilms than *Y. pseudotuberculosis*, due to a sharp increase in the cytoplasmic concentration of c-di-GMP, which activates the HmsHFRS complex (24). This increase results from gene loss during the emergence of *Y. pestis* (24–28). In *Y. pseudotuberculosis*, cyclic-di-GMP is produced by three diguanylate cyclases (DGC: HmsT, HmsD, and YPO2559) and hydrolyzed by three phosphodiesterases (PDEs: HmsP, Rtn, and Y3389) (28–31). By contrast, in *Y. pestis*, two of the three PDEs encoding genes have become pseudogenes, leaving HmsP (PDE1) as the only functional phosphodiesterase. Of note, only two of the three diguanylate cyclases (DGCs: HmsT and HmsD) are functional in *Y. pestis* and are responsible for the synthesis of c-di-GMP (26). In *Y. pseudotuberculosis*, the expression of DGCs is negatively regulated by the Rcs phosphorelay system, a two-component sensory signaling system (RcsB/RcsA) that modulates the transcription of genes involved in cyclic di-GMP synthesis (27). In contrast, in *Y. pestis*, the *rcsA* gene has become a pseudogene, leading to the derepression of *hmsT*, as well as the activation of *hmsD* (24, 32). It is important to note that fleas are not dead-end hosts for *Y. pseudotuberculosis*, which is able to colonize the hindgut. Bacterial key innovations, such as *ure* pseudogenization or *ymt* acquisition, enabled the exploitation of previously unoccupied ecological niches: the flea midgut and proventriculus. This paved the way to adaptive radiation (e.g., in *hms* regulon), increasing the rate of vector transmission through biofilm formation/blockage (28, 33).

In *Y. pestis*, the switch to a vector-borne lifestyle required adaptation to alternating invertebrate and mammalian hosts, each imposing distinct environmental cues. Among them, temperature emerged as a key regulator of exopolysaccharide production. Below 30°C, as in the flea vector, the diguanylate cyclase HmsT is overproduced via increased *hmsT* transcription (25). In contrast, the mammalian host’s higher body temperature promotes HmsT degradation, while HmsP-mediated c-di-GMP hydrolysis persists, resulting in reduced intracellular c-di-GMP levels and inhibition of biofilm formation, which is dispensable during systemic infection.

### Evolution from enteric to lymphatic transmission pathway

In addition to the aforementioned adaptations to vector transmission, the ancestor of *Y. pestis* switched from enteric to systemic transmission through changes in the expression of adhesion and invasion genes. Specifically, the chromosomal *inv* and plasmid-encoded *yadA* genes (encoding factors required for intestinal colonization and invasion by *Y. pseudotuberculosis*) are inactivated in *Y. pestis* through an insertion sequence and a frameshift mutation, respectively (34, 35). By contrast, the type III secretion system, allowing the delivery of effector proteins (Yops) across eukaryotic cell membranes, is conserved throughout the *Yersinia* genus, although expressed by the pYV plasmid, that can be lost *in vitro*. Yop effectors were maintained as they are involved in host immunomodulation and thus required for *Yersinia* spp. transmission to vertebrates (36).

pPCP1 plasmid (9.6 kb) holds three genes: *pst*, encoding pesticin, a bacteriocin targeting the peptidoglycan of competing bacteria (7, 37, 38); *pim*, which protects *Y. pestis* from its own pesticin (38); and *pla*, encoding a plasminogen activator that also exhibits adhesin activity (39), fibrinolytic activity above 37 °C, and plasma coagulase activity below 28°C. Pla is a broad-spectrum protease that promotes dissemination of *Y. pestis* from the skin to the draining lymph node by degrading fibrin clots, the extracellular matrix, and the basement membrane (40, 41). While Pla is essential for escape from the dermal site of infection, it is not required for subsequent systemic spread (41). Thus, the acquisition of *pla* was a key event in the emergence of bubonic plague.

A second plasmid involved in plague pathogenesis is pMT1. It harbors the *caf* operon (*caf1M1A1*), which encodes a proteinaceous pseudo-capsule that confers resistance to phagocytosis (42). Although not essential for virulence, this structure enhances terminal bacteremia and increases the incidence of bubonic plague following flea-borne transmission, thereby promoting the flea-to-mammal transmission cycle (43, 44). Expression of *caf* operon is tightly regulated by temperature, supporting transcriptional adaptation during transition from flea (<28°C) to mammalian (37°C) hosts (45). The plasmid also carries *ymt* and *ypmt1.66c* (encoding a putative helicase), which contribute to host survival and dissemination to the lymph nodes (11, 46).

Altogether, data gathered from genomic analyses and reverse genetics experiments strongly support that the emergence of *Y. pestis* results from pseudogenization, plasmid acquisition, and point mutations conferring efficient vector-borne transmission to an enterobacterial progenitor.

## PLAGUE, AN ANCIENT ZOONOSIS

### Yersinia pestis emergence and discovery

Genomic dating of historical material has notably improved our understanding of *Y. pestis* emergence. While genomic data using coalescent approaches place the emergence of *Y. pestis* around 6,000 years ago, the first pandemic historically documented did not occur until the Justinian Plague (541–544 CE) (47). While we know that the pandemic emerged in the Egyptian port of Pelusium in 541, its ultimate origins are still unknown and may have been associated with Central Asia, contrary to contemporaneous assertions about its “Ethiopian” origins (48). In the course of the period between 541 and 544, plague would spread all over western Eurasia and North Africa, with recurrent waves, on a more limited geographic scale, occurring in the subsequent 200 or so years (49).

The Second Plague Pandemic commenced in Central Asia, likely in the Tian Shan region in the 1330s, spreading between 1338 and 1346 westward into Crimea and then all over western Eurasia and North Africa (1347–1353) (2, 50). Hereafter, plague recurred in dozens of waves radiating from different local Eurasian foci, commencing with the *pestis secunda* of 1356–1366 in south-central Germany; while plague would disappear from western Europe by 1744, it would persevere in the Ottoman Empire for another hundred or so years (51, 52). Undoubtedly, anthropogenic factors such as international trade and warfare in the globalizing world played a paramount role in spreading the disease across regions (53, 54).

The Third Pandemic commenced in Hong Kong in 1894, although its origins are still not clear. Before reaching Hong Kong, it spread in different parts of China, having entered Yunnan in 1772, possibly originating from the Tibet-Gansu region (55, 56). From Hong Kong, it dispersed to Calcutta in 1895 and Bombay in 1896, and then in the course of the next 7 years, entered over 70 ports on 5 continents—all but Antarctica—via infected rodents and their fleas present on ships. The bacillus responsible for the disease was discovered during the Hong Kong epidemic of 1894 by the microbiologist Alexandre Yersin (57–59). By isolating and observing the bacteria under the microscope, and also conducting experimental infection on mice and rats, Yersin was able to confirm its role as the etiologic agent of plague (59). In 1898, Simond demonstrated the involvement of fleas in the transmission of *Y. pestis* (60).

### Wild or sylvatic plague cycle

**Fig 1:**
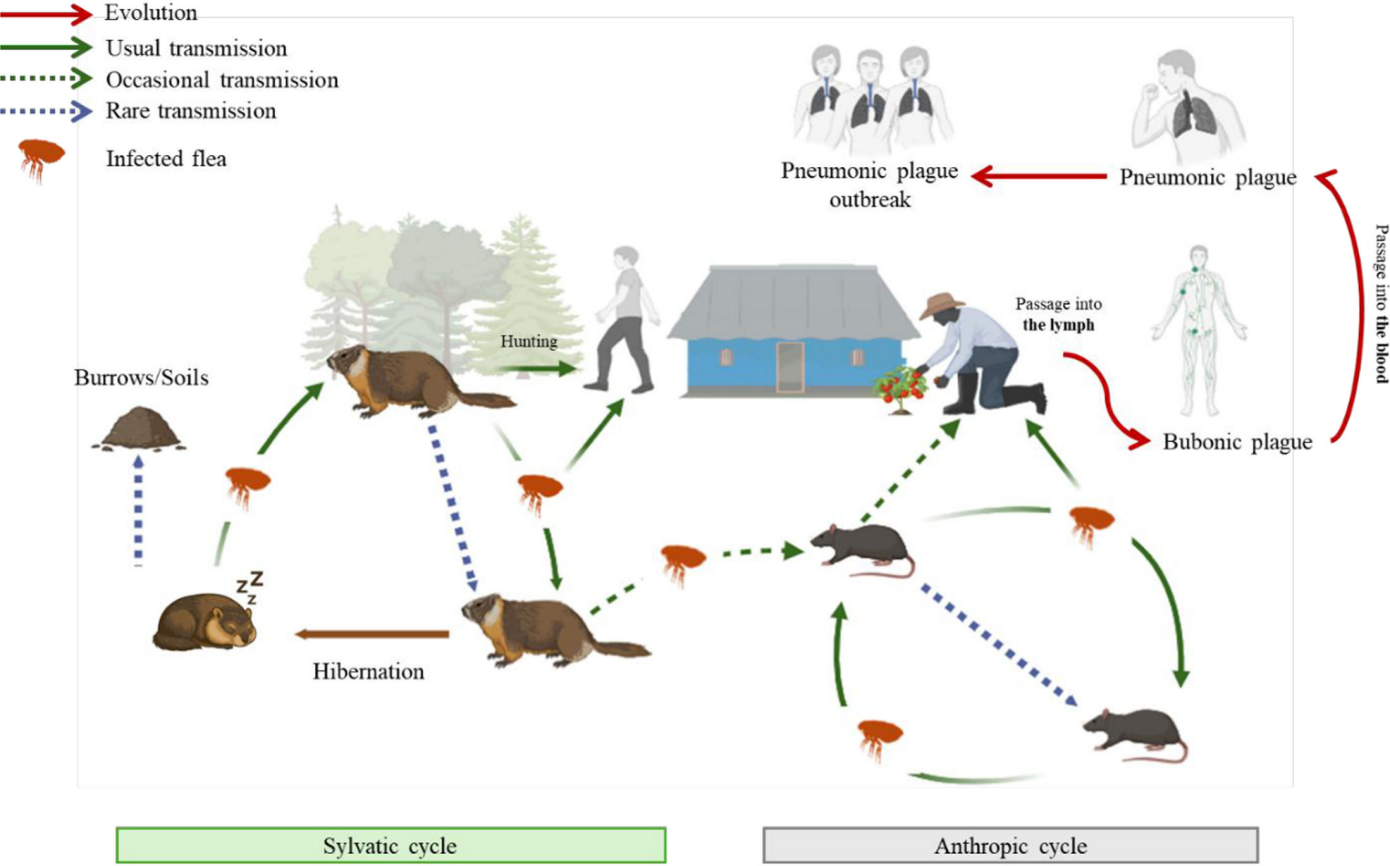
Wild sylvatic and anthropic cycles of plague in small mammals.

From the late 19th century, although *Y. pestis* was known to affect humans via infected fleas of commensal rats and its dispersal was clearly associated with anthropogenic activities, Zabolotnyi, a Ukrainian epidemiologist, hypothesized that wild (non-commensal) rodents were the main actors of maintenance of *Y. pestis* in a number of regions of the Russian Empire and beyond, where plague was endemic (61). The idea of “sylvatic plague” was subsequently pursued by Jorge, a Portuguese scientist, on wild plague reservoirs in the Brazilian “selva” (62). This theory was widely explored during four decades (1926–1960) in former Zaire (now the DR Congo) where not only the murine sylvatic, murine domestic, and human components were thoroughly studied (including the notion of competent reservoirs and vectors) but also six possible modes of transmission were described (63). Furthermore, researchers such as Burroughs (64) and Holdenried (65) in the USA, Macchiavello (66–68) in South America, and Kalina (69), Fenyuk (70), and Bibikov (71, 72) in the former Soviet Union contributed to the in-depth study of natural plague foci, in particular by analyzing its role in the onset of human epidemics (62). These investigations led to the general universal—potentially reductive—concept that *Y. pestis* is sustained by two transmission cycles: the “wild sylvatic cycle,” in which the bacterium is maintained in the local environment by wild mammals, primarily rodents, and their ectoparasites, and the “anthropic cycle,” where the bacterium is transmitted by commensal rodents and their ectoparasites during an epidemic (Fig. 1). Of note, sylvatic plague results from the introduction of *Y. pestis* in virgin ecosystems following human pandemics (reverse transmission concept)—as it happened in some parts of Eurasia in the wake of the Black Death wave (1347–1353) (52, 73), and globally during the last pandemics (55). This sharply contrasts with the classic animal-anthropogenic view of zoonoses, where human diseases mostly emerge from wildlife (74).

According to Burdelov (75, 76), the circulation of *Y. pestis* in natural foci is based on three main processes: preservation, accumulation, and dissemination. *Y. pestis* is firmly established in ecosystems with suitable animal reservoirs, including primary, secondary, and tertiary hosts, each with a specific role in maintaining and transmitting the bacterium. Some species have been characterized as resistant (e.g., *Meriones persicus*) and appear to be primary hosts, while other species appear more susceptible (e.g., *Microtus vinogradovi*) and would be best considered secondary or tertiary hosts (77, 78). Secondary hosts, such as black rats (*Rattus rattus*), although more susceptible than primary hosts, are often more numerous, have colonized many portions of the world through human intervention, and serve as a bridge between wildlife and other species like humans, allowing transient multiplication of the bacterium. Tertiary hosts (e.g., *Homo sapiens*) participate incidentally in the transmission cycle but may be severely affected during outbreaks. To date, 351 mammalian species have been described as playing a potential role in the circulation of *Y. pestis* (78). The bacterium depends on competent vectors (mainly fleas) to enable its inter- and intraspecific transmission. Approximately 80 species of small mammal fleas have been identified as effective vectors, ensuring transmission between hosts (33). Of note, other arthropods such as body lice and human fleas have been proposed to be involved in inter-human transmission (79, 80).

The epidemic manifestations of plague vary considerably among countries. *Y. pestis* can circulate in the form of epizootic cycles (regular outbreaks), enzootic cycles (maintenance in wildlife and/or the environment with sporadic outbreaks), or quiescent phases (persistence in wildlife without human cases for extended periods). Madagascar has outbreaks of epizootic plague with seasonal epidemics occurring every year since 1921. Conversely, other regions experience sporadic epidemics at prolonged intervals at the level of decades. This is notably the case in Algeria, where an epidemic flared up in 2003 then 2008, after some 50 years of latency (81, 82), or in the Atyrau district of West Kazakhstan, where no plague was recorded between 1925 and 1986 (83). Work by Neerinckx et al. (84), comparing various plague outbreaks, has revealed the existence of both environmental and biotic similarities between plague areas. Indeed, specific climatic conditions, such as a humid climate, cooler temperatures, and higher altitudes, are common to the affected regions. The occurrence and abundance of hosts, vectors, and bacteria are also determining factors. The influence of these elements varies from one plague focus area to another, modulating the emergence of epidemics. In addition, movements of hosts and vectors can be influenced by climate—in particular, dry-wet cycles duration—or season, as well as by anthropological activities including agricultural practices, which, for example, in Tanzania have led to an increase in rodent populations within plague foci (85).

A large body of evidence has thus shown that human trade was key in *Y. pestis* worldwide dispersal, while the diversity of the ecosystems able to support bacterial persistence suggests an outstanding plasticity of this pathogen.

## PLAGUE CASES REPORTED IN THE WORLD

**Fig 2:**
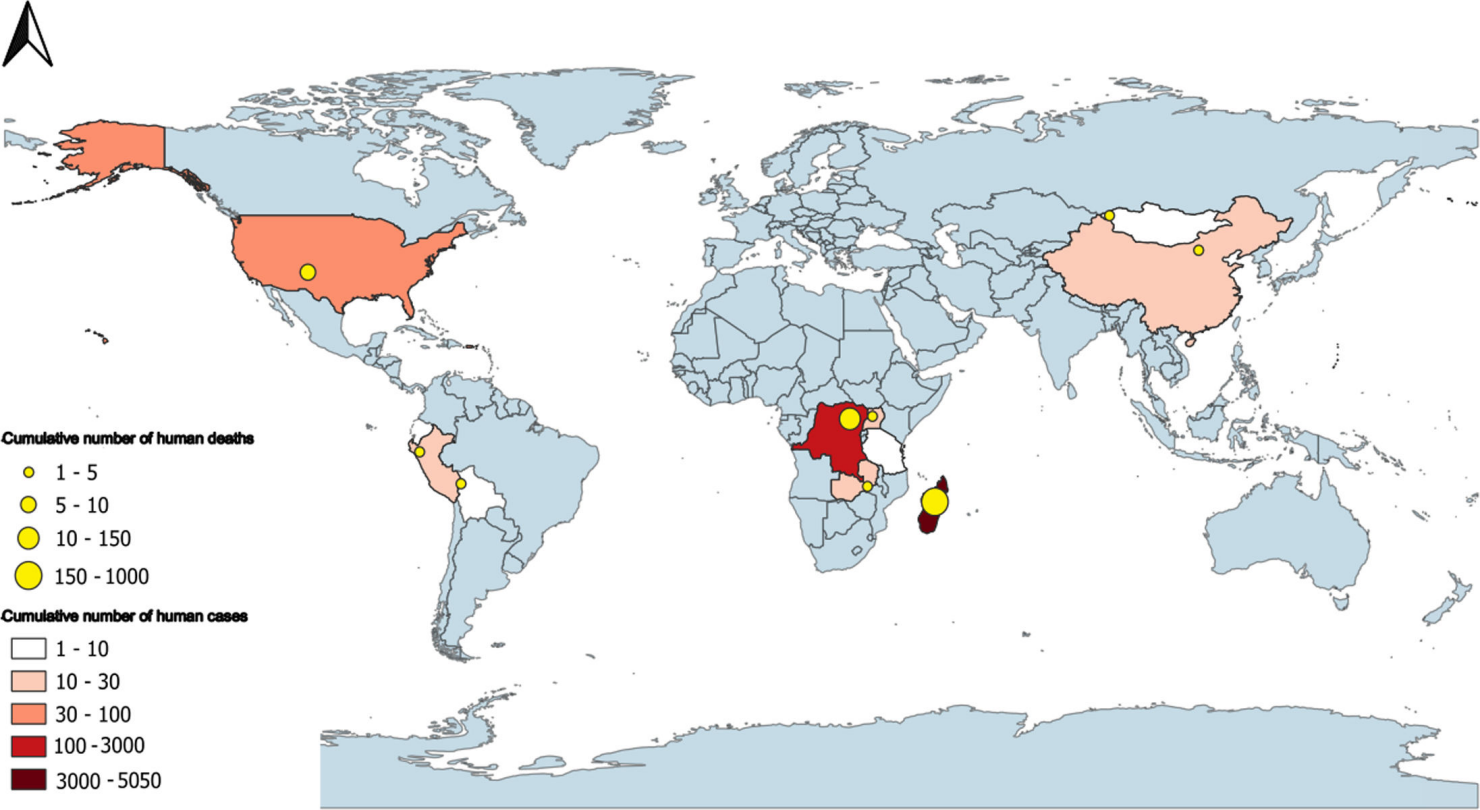
Distribution of human plague cases worldwide between 2014 and 2024. The map was created using QGIS 3.34.2 software.

Between 2014 and 2024, 11 countries reported sporadic cases or epidemics of plague (Fig. 2). Madagascar remains the most affected endemic area, with an annual incidence typically ranging between 100 and 500 cases (86). A major outbreak in 2017, resulting in 2,676 recorded cases and 238 deaths, is exceptional (87). Other African nations, such as the DR Congo, Uganda, Zambia, and Tanzania, have reported cases, mainly in rural areas (88, 89). In Asia, since 2013, plague outbreaks were only observed in China (Inner Mongolia), Kyrgyzstan, and Mongolia, where infections were often linked to the consumption of marmot meat (90) or direct contact with these animals. In the USA, there have been sporadic reported cases every year since 2000, with an average of about five cases per year, particularly in western states such as Arizona, Colorado, and New Mexico (91). In South America, Peru has recorded the highest number of sporadic cases over the past 20 years (92), and a few cases have also been reported in Bolivia and Ecuador (93). These regions, although ecologically diverse—ranging from desert areas to tropical forests—share common characteristics such as seasonal alternation between dry and wet periods, high altitude, and the presence of plateaus or semi-arid zones.

### Direct impacts of anthropogenic activities on *Y. pestis* distribution

In addition to long-distance maritime and overland trade, which may have been responsible for the spread of plague at a global scale, local trade has had a paramount impact on the epidemiology of the disease. In Madagascar, where *Y. pestis* was originally confined to coastal areas (94), the construction of a train line connecting the main eastern harbor of Toamasina to the capital, Antananarivo, facilitated the establishment of *Y. pestis* in the Central Highlands (94). A similar situation prevailed in East Africa with the construction of the British Uganda Railway in the 1890s, connecting Lake Victoria with the Swahili coast (95). In the DR Congo, following the 1926 and 1928 emergence of plague, control measures were strengthened through vaccination campaigns and the culling of reservoirs and control of vectors (63). However, post-independence turmoil from the 1960s onward disrupted these efforts, enabling the expansion of the Ituri focus, the establishment of new hosts, and vector and rodent population densities increases. Between 2004 and 2014, more than 4,600 cases, including 349 fatalities, were reported from that country (96).

Meanwhile, on the Hulun Buir plateau, located on the China-Mongolia border, plague was highly prevalent in the early 20th century, linked to the hunting of marmots for their meat and fur. Between 1923 and 2019, plague cases became rare due to the drastic decline in marmot populations (97). Following preventive restrictions on marmot hunting implemented by the Chinese government, *Marmota sibirica* began repopulating the Chinese autonomous region, and over 20 human cases of plague were observed once hunting reopened (97, 98). Domestic animals, due to their close contact with humans, can also serve as sources of plague outbreaks (99, 100). A dog was involved in triggering an outbreak of pneumonic plague in July 2009 in Qinghai Province, China (100). Plague infection in Tibetan sheep (*Ovis aries*) has been reported in the same province since the 1950s (101).

### Hosts and vectors: some knowns

Epidemiological data collected over the past decade in African and South American countries affected by plague present several limitations, including geographic coverage, specifically not complete coverage of at-risk areas, infrequent investigations, underreporting, and the use of poorly comparable diagnostic methods. Most epidemiological studies on wildlife have relied on observational, serological, and molecular approaches, although using *pla* alone, which has been shown to be non-specific to *Y. pestis*, thus calling for cautious interpretations (102).

Whether in Zambia, Uganda, Tanzania, Madagascar, or the DR Congo, *Rattus* genus, an introduced rat originally from Asia, plays a major role in the emergence and persistence of plague within these endemic zones (103–111). Local rodents, *Mastomys* genus, have been confirmed as involved in Zambia (103), DR Congo (63), and Tanzania. Other peri-urban rodents, such as mice (*Mus*), participate in the circulation of the bacterium in Madagascar (108, 110) and Tanzania (107). Shrews (*Crocidura*), though phylogenetically distinct from rodents, have been positively associated with plague outbreaks in Zambia (103) and Uganda (109). Meanwhile, *Suncus murinus*, another genus of shrew, has been documented in the transmission of *Y. pestis* in Madagascar. Other genera of native rodents (Table 1) are also considered potential hosts of *Y. pestis* in Tanzania, DR Congo, and Uganda, such as *Arvicanthis*. Finally, other wild small mammals, such as *Tenrec ecaudatus*, endemic to Madagascar, have shown evidence of exposure to *Y. pestis* (112). The main flea vectors in Africa include cosmopolitan (*Xenopsylla cheopis*, *Xenopsylla brasiliensis*, and *Pulex irritans*) and endemic (*Dinopsyllus lypusus*, *Ctenopthalmus* spp., and *Synopsyllus fonquerniei*) (63, 106, 113–116).

**TABLE 1:** Ecological and epidemiological characteristics of endemic plague foci (2014–2024)

Country	Year and place of first plague report	Localization of plague foci	Characteristic(s) of plague foci	Vertebrate hosts	Vectors	No. of cases (2014–2024)	Last reported epidemics	References
Democratic Republic of the Congo	1926, in the Ituri Mountains near Lake Albert	Ituri, Haut-Uele Districts, North Kivu Province	Mountainous area located at around 1,000 m with a dry tropical climate.	*Arvicanthis abysinnicus*, *Cavia porcellus*, *Lemniscomys striatus*, *Mastomys natalensis*, *Mus minutoides*, and *Rattus rattus*	*Pulex irritans*, *Xenopsylla cheopis*, *Xenopsylla brasiliensis*, *Dinopsyllus* spp., and *Ctenophthalmus* spp.	2,429 cases and 130 deaths	February 2024, 50 cases and 5 deaths, Ituri	(63, 84, 96, 117–119); Ministry of Health, unpublished data
Madagascar	1898, in the port of Toamasina	Central Highlands (Analamanga, Bongolava, Itasy, Vakinankaratra, Amoron'i Mania, Haute Matsiatra Regions) North (Sofia and DIANA Regions), and East (Atsinanana Region) of the country	Madagascar’s Central Highlands (above 1,000 m), tropical montane climate with moderate temperatures and dense forests. The north (0–2,876 m), subequatorial climate, dry in the northwest, humid in the northeast, with forests, grasslands, and agricultural zones. The east (0–1,500 m), humid and tropical, dominated by humid forests, montane forests, and mangroves.	*Mus musculus*, *Rattus rattus*, *Rattus norvegicus*, *Suncus murinus*, and *Tenrec ecaudatus*	*Synopsyllus fonquernei* and *Xenopsylla cheopis*	5,036 cases and 680 deaths	2023, 57 cases	(87, 94, 108, 110, 112, 113, 118, 120–127)
Uganda	1949 in the south of the country	West Nile region, Arua, and Okora Districts	Increased risk during the dry season and when farmlands are fallow. Plague foci are located at altitudes of 1,300 m.	*Arvicanthus niloticus*, *Crocidura* sp., and *Rattus rattus*	*Xenopsylla brasiliensis* and *Xenopsylla cheopis*	11 cases and 1 death	February 2019, 2 cases and 1 death, Zamboro District	(109, 118, 128–132)
Tanzania	1980 in Mkunki, Lushoto District	Usambara Mountains, Lushoto District	Altitude between 900–2,250 m, in agricultural areas, and soils rich in iron, manganese, and magnesium.	*Arvicanthis nairobae*, *Graphiurus murinus*, *Lemniscomys striatus*, *Lophuromys flavopunctatus*, *Mastomys natalensis*, *Mus minutoides*, *Otomys* sp., *Praomys delectorum*, *Rattus rattus*, and *Tatera* sp.	*Dinopsyllus lypusus*, *Pulex irritans*, *Xenopsylla cheopis*, and *Xenopsylla brasiliensis*	2 cases	December 2019, two suspect cases, Babati District	(104, 105, 107, 111, 115, 118, 133–140)
Zambia	1917 in the Chama District	Namwala, Sinda, Chama, Lundazi, and Zambezi districts	Altitude between 900–1,300 m, in agricultural areas, and semi-arid climate.	*Canis* sp.*, Crocidera* sp., *Mastomys sp., Rattus* sp., and *Tatera* sp.	*Xenopsylla* sp.	21 cases and 3 deaths	2015, 21 cases, 3 deaths, Nyimba	(89, 103, 118, 139)
Mongolia	1897	Western part	Altitudes ranging from 640 to 3,500 m, and mountainous steppe zones.	*Allactaga sibirica*, *Alticola argentatus*, *Alticola strelzovi*, *Cardiocranius paradoxus*, *Lasiopodomys brandti*, *Marmota baibacina*, *Marmota sibirica*, *Meriones meridianus*, *Meriones unguiculatus*, *Microtus gregalis*, *Mustela altaica*, *Mustela eversmanni*, *Oenanthe oenanthe*, *Ochotona daurica*, *Ochotona pallasi*, *Phodopus sungaris*, *Rhombomys opimus*, *Spermophilus erythrogenys*, *Spermophilus undulatus*, and *Vulpes corsac*	*Oropsylla silantiewi*	13 cases and 6 deaths	2023, 1 case	(118, 141–144)
China	5th century BC, Inner Mongolia	Inner Mongolia, Qinghai, Gansu, Tibet, Sichuan, and Xinjiang	Altitudes ranging from 1,000 to 1,500 m, mountainous steppe zones.	*Marmota sibirica*, *Meriones unguiculatus*, *Microtus brandti*, and *Spermophilus dauricus*	*Nosopsyllus laeviceps kuzenkovi*	17 cases and 7 deaths	2023, 3 cases	97, 98, 118, 143, 145)
Bolivia	1921 in Padcaya	La Paz and Santa Cruz regions	Andean valleys (1,800–3,000 m), tropical and subtropical moist broadleaf forests, grasslands, savannas, and shrublands. Montane grasslands and shrublands.	*Calomys boliviae*, *Calomys venustus*, *Dasyprocta azarae*, *Galea littoralis littoralis*, *Oligoryzomys flavescens*, *Oxymycterus paramensis*, *Rattus rattus*, *Sylvilagus brasiliensis*, and *Tapecomys wolffsohni*	*Pulex irritans* and *Xenopsylla cheopis*	3 cases and 1 death	2018,1 case, 1 death	(93, 118, 146–149)
Ecuador	1908 in the port of Guayaquil	Chimborazo and Cotopaxi regions	Andean valleys (2,500–3,800 m), tropical and subtropical dry broadleaf forests. Montane grasslands and shrublands.	*Cavia porcellus*, *Oligoryzomys flavescens*, *Oligoryzomys longicaudatus*, *Phyllotis andium*, *Rattus rattus*, *Sigmodon peruanus*, *Simosciurus nebouxii*, and *Sylvilagus brasiliensis*	*Pulex irritans*	1 case	2021, 1 case	(93, 118, 147–150)
Peru	1903 in the port of Callao, on Peru’s central coast	Cajamarca, La Libertad, Lambayeque, and Piura regions	Andean valleys (455–3,130 m), mountain meadows and shrublands, deserts and xerophytic shrublands, and with little precipitation.	*Akodon dolores*, *Akodon orophylius*, *Calomys expulsus*, *Cavia porcellus*, *Cerradomys angguthi*, *Galea spixii*, *Hylaeamys perenensis*, *Monodelphis domestica*, *Necromys lasiurus*, *Oecomys sp.*, *Oligoryzomys nigripes*, *Rhipidomys leucodactylus*, *Rattus rattus*, *Simosciurus nebouxii*, and *Thrichomys laurentius*	*Polygenes sp.*, *Pulex irritans*, *Tiamastus* sp., and *Xenopsylla cheopis*	24 cases and 1 deaths	2024, 2 cases, Cajarmaca	(92, 93, 118, 147–149, 151, 152)
United States of America	1900 in San Francisco, California	Dakota, Nebraska, Kansas, New Mexico, Oklahoma, and Texas	Rocky Mountains, clayey or sandy soils, rich in iron, with variable annual precipitation ranging from 500 to 2,500 mm	*Canis latrans*, *Canis lupus*, *Castor canadensis*, *Cynomys ludovicianus*, *Gulo gulo*, *Lynx rufus*, *Mephitis mephitis*, *Procyon lotor*, *Puma concolor*, *Sus scrofa*, *Sylvilagus* sp., *Taxidea taxus*, *Urocyon cinereoargenteus*, *Ursus americanus*, *Vulpes macrotis*, *Vulpes velox*, and *Vulpes vulpes*	*Oropsylla montana* and *Xenopsylla cheopis*	54 cases and 8 deaths	2024, 2 cases, 1 death, New Mexico	(91, 118, 153–157)

Latin America has very limited plague epidemiological data related to wildlife. In Bolivia, Peru, and Ecuador, most species considered potential hosts are rodents belonging to the endemic family Cricetidae (Table 1). Various genera belonging to different rodent families are also considered potential hosts, including *Galea*, *Dasyprocta*, *Trichomys*, and *Rattus*. Lastly, other mammals such as marsupials (*Monodelphis domestica*) and lagomorphs (*Sylvilagus brasiliensis*) have been suggested as hosts (147, 149, 152). Guinea pigs (*Cavia porcellus*) are experimentally susceptible to plague and can meet the criteria for amplifying hosts, especially in regions where they live in close association with humans, particularly in the Andean regions or DR Congo, where they are kept domestically (119, 158). In Latin America, the main flea vectors include *X. cheopis*, *Polygenis* sp., *Tiamastus* sp., and *P. irritans* (147).

In the USA, Gunnison’s prairie dogs (*Cynomys ludovicianus*) are *Y. pestis* primary hosts (159–162). Carnivores in plague foci areas are also frequently exposed to the bacterium either by feeding on infected animals or through bites of infected fleas. Some large predators, such as *Puma concolor* (163) or *Lynx canadensis* (164), are susceptible to the infection and die. In contrast, other species, such as *Canis latrans* and *Vulpes* spp., survive the infection and generally show mild or no clinical signs (154, 157, 165). A similar pattern is observed in Mongolia, where carnivore species have shown immunity to *Y. pestis*, likely acquired through feeding on infected rodents (141). Although numerous flea species found in the USA have demonstrated competence in transmitting *Y. pestis* under laboratory conditions (166), *Oropsylla montana* and *X. cheopis* are considered the main vectors (167).

While plague foci with endemic transmission cover nearly one-third of Mongolia, China reports 15 plague foci, 4 of which are in the Inner Mongolia Autonomous Region and are characterized by a specific combination of host/vector species and *Y. pestis* ecotypes (141, 168, 169). The main primary hosts in both countries are marmots, but other rodent species have also been shown to be involved, such as pikas (*Ochotona*), ground squirrels (*Spermophilus*), hamsters (*Phodopus*), voles (*Alticola*, *Lasiopodomys*, and *Microtus*), gerbils (*Meriones* and *Rhombomys*), or jerboas (*Allactaga* and *Cardiocranius*; Table 1) (141). The main flea vector appears to be *Oropsylla silantiewi* that parasitizes marmots (141).

Other countries, such as Kazakhstan, Armenia, and Georgia, have plague-endemic areas but have not reported any human cases in the past 10 years. In Kazakhstan, the main established hosts of *Y. pestis* are the great gerbil (*Rhombomys opimus*), ground squirrels (*Spermophilus* spp.), and marmots (*Marmota* spp.) (170–172). On the Transcaucasian plateau, the primary hosts are voles (*Microtus vinogradovi* and *Microtus arvalis*) (173), and the main flea vectors are *Xenopsylla* sp. and *Nosopsyllus* sp.

While several studies have been published regarding the competent vectors and reservoirs in Central Asia and North America, African and Latin American plague foci suffer from a lack of comprehensive data sets.

## HYPOTHESES REGARDING MAINTENANCE OF *YERSINIA PESTIS* IN THE ENVIRONMENT

The mechanism of how *Y. pestis* is maintained in the environment remains an open question. This is particularly challenging in plague foci where successive outbreaks are caused by distinct bacterial lineages (174). An older hypothesis suggests the existence of silent animal reservoirs (such as hibernating species), notably certain species of asymptomatic wild rodents. Another hypothesis suggests prolonged survival of the bacterium in soils with suitable microclimatic conditions of humidity and temperature. Furthermore, recent studies suggest that *Y. pestis* may transit via free-living protozoa or amoebae, acting as temporary environmental hosts. Finally, it has been suggested that certain arthropods may act as discrete reservoirs.

### Role of mammals with reduced metabolism and *Yersinia*-resistant mammals

The discrete reservoir hypothesis suggests that *Y. pestis* survives in the environment via isolated, specific reservoirs that maintain the microbe for months. The bacteria, for which growth is inhibited at temperatures below 10°C, can remain dormant in a hibernating host, such as Himalayan marmots (*Marmota himalayana*), whose metabolism and immune system are slowed down (90, 175, 176). Upon awakening, marmots become susceptible to the infection as the body temperature is at non-hibernating levels, with bacterial growth leading to the onset of the disease, and in certain cases death, in this mammal (177). One hypothesis holds that plague outbreaks may be associated with bio-ecological stresses originating in the context of a complex tripartite process during marmot hibernation, consisting of the “bacteremic,” “adaptive,” and “mutative” phases. If bacteria are unable to adapt during the process, they may experience stress-induced mutagenesis, whereby they mutate, resulting in outbreaks first in primary, then in secondary hosts (178).

In the USA, prairie dogs (*Cynomys*) have been identified as the primary species affected by *Y. pestis*, which has contributed to the decline of certain populations. In the Moreno Valley, *C. ludovicianus* populations were severely affected by *Y. pestis* and declined from 100,000 to 250 individuals between 1984 and 1987 (159–162). This led to the decline of its predator, the black-footed ferret (*Mustela nigripes*), resulting from both the disease and a shortage of prey (179). To cope with winter conditions, these rodents exhibit physiological adaptations: *C. leucurus* enters torpor, while *C. ludovicianus* remains active (180) as long as there is no shortage of food or water. Reduced metabolism of prairie dogs during the winter season may facilitate the establishment or persistence of *Y. pestis*.

*Rattus* and *Mastomys* play a key role in plague epidemiology as they facilitate *Y. pestis* transmission to humans. Certain individuals of *R. rattus* survive infection and subsequently develop immunity to the bacterium (110). *Mastomys natalensis* is widely distributed across the African continent and divided into genetically distinct geographically structured subpopulations (181). These intraspecific variations may lead to differing levels of susceptibility or resistance to *Y. pestis* infection, thereby shaping local transmission dynamics (181). These two genera of rodents, which play a limited role in the transmission cycle, are considered secondary hosts with resistance varying according to the endemic presence of *Y. pestis*. Resistant populations of *Rattus* and *Mastomys*, found in endemic areas, may have adapted, thereby promoting increased circulation of the bacterium and a higher risk for humans.

### Maintenance in soil

The telluric maintenance hypothesis was first tested by Yersin himself in 1894 following the isolation of an attenuated *Y. pestis* strain in the soil 4–5 cm below the surface of a house sheltering plague victims in Hong Kong (182). He then sampled soils from other plague-affected homes and demonstrated the presence of the bacterium up to 30 cm below the soil surface. However, the persistence of *Y. pestis* in the environment remains poorly documented. In the USA, a study demonstrated that *Y. pestis* could persist in the environment for up to 21 days after the death of an infected puma. Indeed, Swiss-Webster strain mice developed plague 12 h after being inoculated with soil samples collected from the area where the puma died (183). Another study showed the persistence of virulent *Y. pestis* in sterile soil for up to 40 weeks (184). In contrast, the probability of transmission between soil and small mammals appeared limited (<1%) under laboratory conditions (185). Importantly, the bacterium is able to maintain itself in the environment—under a defined set of microclimatic conditions—without a host and persist for up to 280 days (184, 186).

Bacterial viability of *Y. pestis* has been demonstrated under controlled temperature and humidity conditions on several different substrates, such as stainless steel, polyethylene, and glass, with rapid decline and no detectable bacteria after 48 h under standard conditions; however, in the presence of a rich culture medium (brain heart infusion [BHI]), a very small number of bacteria may persist up to 3 days (187). Viability has also been observed in water microcosms under viable but non-cultivable conditions for 28 days at 4°C (188). *Yersinia pestis* can switch to an attenuated form, known as “L-form,” under environmental stress and survive in mammals and arthropods for several months (189, 190). Some investigations have suggested that soil salinity might contribute to the persistence of *Y. pestis* reservoirs, partly due to the ability of the bacterium to survive and replicate within soil amoebae and to the development of halo-tolerance (5, 183, 184, 190–192). Another study linked the presence of *Y. pestis* reservoirs in the USA to saline and arid soil conditions (5). A study by Stenseth and colleagues (193) found that current *Y. pestis* reservoirs in China and North America are located in areas with specific soil characteristics—particularly higher levels of cadmium, copper, iron, magnesium, sodium, antimony, and uranium, along with more alkaline soil pH and higher clay content. In contrast, soils with elevated concentrations of calcium, cerium, molybdenum, and yttrium, as well as (more acidic) pH, were less likely to support *Y. pestis* reservoirs.

Other research carried out in various plague zones in Asia (Russia, Kazakhstan, China, and Mongolia) revealed a positive association between the presence of the bacterium, cobalt, and manganese and a negative association associated with copper, zinc, and molybdenum (194). In China, a comparative study of the mineralogical and organic composition of soils collected from the burrows of *Y. pestis* hosts (e.g., marmots) and non-hosts mammals showed lower levels of organic matter and alkaline-hydrolyzable nitrogen, alongside higher electrical conductivity and total soluble salt content in habitats occupied by marmots. Moreover, these soils exhibited elevated concentrations of nickel, chromium, and iron but reduced levels of zinc and selenium. Consequently, soil microbial diversity was found to be significantly lower in marmot burrows, likely associated with the specific physicochemical properties of these soils (195).

### Survival in amoebae

The genus *Acanthamoeba*, a free-living ubiquitous amoeba, has been identified as a host or reservoir for numerous pathogenic bacteria (196). Amoebae protect bacteria in the dormant (cyst) phase and facilitate their proliferation in the trophozoite phase, thereby enhancing their ability to infect human and animal hosts. These interactions also favor the emergence of new pathogenic strains through genetic exchange within this cellular environment (197). Under laboratory conditions, *Y. pestis* is capable of surviving within soil amoebae, such as *Acanthamoeba castellanii* (up to 5 days) and *Dictyostelium discoideum* (up to 2 days), while retaining its intracellular mechanisms necessary for infecting mammalian macrophages (191, 192). These observations highlight the need to examine amoeba-*Y. pestis* interactions to better understand the mechanisms of pathogen persistence and transmission in different environmental and clinical contexts.

### The role of invertebrates

The persistence of *Y. pestis* during inter-epizootic periods appears to be closely linked to the longevity of flea vectors in the burrows of hibernating rodents, particularly the marmot (*Marmota baibacina*). Studies have shown that infected fleas, such as *Oropsylla silantiewi* and *Rhadinopsylla li ventricosa*, can survive in uninhabited marmot burrows for 8–14 months (198, 199). Laboratory experiments confirmed that *Y. pestis* can persist almost a year within *O. silantiewi* fleas (200). The natural sealing of burrows by marmots before hibernation stabilizes internal microclimates and appears to enhance flea survival by providing protection against cold and desiccation (201). According to one hypothesis, during the “bacteraemic” phase of hibernation, marmot flea larvae transfer from the fur to the oral cavity, where they feed on its mucous membranes and on blood (178). This behavior may thus contribute to the long-term maintenance of *Y. pestis*-infected fleas in the environment.

Most importantly, it has been recently shown that *Y. pestis* can be transmitted transovarially in *X. cheopis*, with 50% vertical transmission to the eggs (202). Such levels of vertical transmission could be sufficient to consider fleas as reservoirs. However, such transmission pathways may need vertebrate hosts to allow horizontal transfer from infected to uninfected fleas, in a way comparable to the horizontal transmission of pathogens between co-feeding ticks (203, 204).

An essential yet under-explored aspect deserves particular attention: the role of the flea microbiota in the infection process. Indeed, this microbiota can influence the bacterium’s ability to colonize the flea’s proventriculus, interfere with the formation of the biofilm necessary for transmission, modify the intestinal environment, and affect its immunity. Thus, the internal microbial composition of fleas could either promote or inhibit infection and the spread of plague. The flea microbiome is largely affected by species and, to a lesser extent, by location and circulates by both horizontal and vertical transmissions (205). In 2015, it was shown that the bacterial composition of fleas depended on flea species, collection site, season, rainfall, altitude, and host species (206). A recent study (207) of fleas (including *Y. pestis* vectors) from Madagascar small mammals demonstrated that bacterial composition varied according to flea species and collection site, but not host species. This pattern suggests that the environment drives the microbial community composition of (detritivorous and blood feeding) larval fleas, which may in turn control the competence of the adult stages.

The presence of *Y. pestis* has also been reported in other arthropods, such as lice and ticks, in Mongolia and DR Congo (117, 141). It has been further shown that *Y. pestis* is capable of multiplying in the human body louse (*Pediculus humanus*) to colonize its Pawlowsky glands (corresponding to salivary glands) and to be transmitted by this arthropod (79, 208).

It appears that most old hypotheses regarding environmental persistence of *Y. pestis* still remain to be tested, while other possible mechanisms more recently proposed deserve to be explored.

## FUTURE CHALLENGES

*Yersinia pestis* is an emblematic pathogen with a peculiar evolution among zoonotic agents. Indeed, by contrast with most emerging diseases that originate from wildlife (74), it appears that humans have been important venues of *Y. pestis* introduction in wild mammal populations (209, 210). As shown here, given the diversity of plague epidemiological contexts throughout the world, superimposed on the number of possible vector or reservoir species, as well as factors such as temporal disease dynamics, the enzootic cycles appear distinct in each of the plague foci. This highlights an important plasticity of *Y. pestis*, which is able to adapt to local environmental conditions, including climate and soil physical properties, as well as variable faunistic aspects of vectors and mammal groups. The recurrence of plague in geographically stable areas after long periods of epidemiological silence suggests the existence of a long-term environmental reservoir. In response to these challenges, several national and international programs have been established to monitor and study plague dynamics across endemic regions (211). Therefore, it is of the utmost importance to implement research and surveillance programs that include molecular and serological testing of wild and synanthropic mammals and vectors, as well as environmental sampling. Such programs will allow new insights on the main actors of *Y. pestis* transmission at play in each specific environmental context. A better understanding of sylvatic plague is required to avoid anthropogenic habitat disturbance that may facilitate the maintenance and replication of *Y. pestis* and improve preparedness to future outbreaks.
